# Capturing lessons learned from evidence-to-policy initiatives through structured reflection

**DOI:** 10.1186/1478-4505-12-2

**Published:** 2014-01-17

**Authors:** Fadi El-Jardali, John Lavis, Kaelan Moat, Tomas Pantoja, Nour Ataya

**Affiliations:** 1 Department of Health Management and Policy, American University of Beirut, Riad El Solh, PO Box 11–0236, Beirut 1107 2020, Lebanon; 2 Knowledge-to-Policy (K2P) Center for Health, American University of Beirut, Riad El Solh, Beirut 1107 2020, Lebanon; 3 Research, Advocacy and Public Policy-making, Issam Fares Institute for Public Policy and International Affairs, American University of Beirut, Riad El Solh, PO Box 11–0236, Beirut 1107 2020, Lebanon; 4 Department of Clinical Epidemiology and Biostatistics, McMaster University, CRL-209, 1280 Main St. West, Hamilton, ON L8S 4 K1, Canada; 5 McMaster Health Forum, MML-417, 1280 Main St. West, Hamilton, ON L8S 4 L6, Canada; 6 Centre for Health Economics and Policy Analysis, McMaster University, CRL-209, 1280 Main St. West, Hamilton, ON L8S 4 K1, Canada; 7 Department of Political Science, McMaster University, CRL-209, 1280 Main St. West, Hamilton, ON L8S 4 K1, Canada; 8 Program in Policy Decision-making, Centre for Health Economics and Policy Analysis, McMaster University, CRL-209, 1280 Main St. West, Hamilton, ON L8S 4 K1, Canada; 9 Department of Family Medicine, Pontificia Universidad Católica de Chile, Centro Médico San Joaquín, Av. Vicuña Mackenna 4686, Macul, Santiago, Chile

## Abstract

**Background:**

Knowledge translation platforms (KTPs), which are partnerships between policymakers, stakeholders, and researchers, are being established in low- and middle-income countries (LMICs) to enhance evidence-informed health policymaking (EIHP). This study aims to gain a better understanding of the i) activities conducted by KTPs, ii) the way in which KTP leaders, policymakers, and stakeholders perceive these activities and their outputs, iii) facilitators that support KTP work and challenges, and the lessons learned for overcoming such challenges, and iv) factors that can help to ensure the sustainability of KTPs.

**Methods:**

This paper triangulated qualitative data from: i) 17 semi-structured interviews with 47 key informants including KTP leaders, policymakers, and stakeholders from 10 KTPs; ii) document reviews, and iii) observation of deliberations at the International Forum on EIHP in LMICs held in Addis Ababa in August 2012. Purposive sampling was used and data were analyzed using thematic analysis.

**Results:**

Deliberative dialogues informed by evidence briefs were identified as the most commendable tools by interviewees for enhancing EIHP. KTPs reported that they have contributed to increased awareness of the importance of EIHP and strengthened relationships among policymakers, stakeholders, and researchers. Support from policymakers and international funders facilitated KTP activities, while the lack of skilled human resources to conduct EIHP activities impeded KTPs. Ensuring the sustainability of EIHP initiatives after the end of funding was a major challenge for KTPs. KTPs reported that institutionalization within the government has helped to retain human resources and secure funding, whereas KTPs hosted by universities highlighted the advantage of autonomy from political interests.

**Conclusions:**

The establishment of KTPs is a promising development in supporting EIHP. Real-time lesson drawing from the experiences of KTPs can support improvements in the functioning of KTPs in the short term, while making the case for sustaining their work in the long term. Lessons learned can help to promote similar EIHP initiatives in other countries.

## Background

In recent years, there has been an increase in global calls to ensure health policymaking is informed by research evidence to improve health systems and population health [1,2]. Most recently, the Beijing Statement from the Second Global Symposium on Health Systems Research called for promoting knowledge translation (KT) by developing communities of practice and enhancing trust between researchers, policymakers, and stakeholders [3].

In response to these calls, efforts have been directed towards strengthening evidence-informed health policymaking (EIHP) in low- and middle-income countries (LMICs) where major health challenges exist and there are limited resources to address these challenges. Particularly, KT platforms (KTPs), which are partnerships between policymakers, researchers, civil society groups, and other key health system stakeholders, are being established worldwide by the World Health Organization Evidence-Informed Policy Networks (WHO EVIPNet) in order to facilitate the process of translating research evidence into policy and action [4]. Some of the activities supported by these KTPs include the synthesis of research to address health policy priorities (e.g., evidence briefs for policy), as well as convening deliberative dialogues (sometimes called policy dialogues) that bring different health actors together to deliberate about a problem, the options for addressing it, and key implementation considerations [5-7].

With KTPs at various stages of implementation, little is known about their activities for enhancing EIHP [7,8]. A recent review of the experience of the Zambia Forum for Health Research (ZAMFOHR) highlighted key lessons learned during the organizational development of the KTP including the importance of organizational issues to achieve buy-in among the community and the necessity of investing in building the capacity of the wider community and KTP staff to undertake KT activities [7]. Few studies exist on KT in LMICs and there are inconsistencies among the available studies on the factors identified as influencing the use of evidence [9,10]. As such, there is a need to understand the factors that enable or hinder KTP work in LMICs in order to improve the ongoing activities of KTPs and inform the experience of other growing KTPs.

This paper seeks to gain a better understanding of i) the activities conducted by KTPs from LMICs to link research evidence to policy, ii) the way in which KTP leaders, policymakers, and stakeholders perceive these activities and their outputs, iii) key facilitators for supporting KTP work, as well as the challenges encountered and the lessons learned that can assist in overcoming these challenges, and iv) factors that can help to ensure the sustainability of KTPs. This paper solicits the views of KTP leaders, policymakers, and stakeholders from 10 KTPs in LMICs. It also draws on a document review and observation of deliberations at the International Forum on EIHP in LMICs held in Addis Ababa from August 28^th^ to 30^th^ 2012, which was hosted by the Ethiopian Health and Nutrition Research Institute (EHNRI), and organized by EVIPNet. The International Forum brought together more than 137 KTP team members, policymakers, stakeholders, and researchers from 25 countries to share their experiences on EIHP and identify opportunities for improving EIHP in LMICs.

## Methods

This structured reflection drew on multiple sources of qualitative data, namely i) semi-structured group and individual interviews with key informants including KTP leaders, policymakers, and stakeholders from the countries where the KTPs are formed, and Knowledge Translation Platform Evaluation (KTPE) team members who were responsible for evaluating KTPs in different countries; ii) document review of the report from the Forum on EIHP in LMICs; and iii) observation of deliberations at the International Forum (Table 1). The approach used in this study was primarily focused on the data gained from the group and individual interviews. Data generated from interviews were triangulated with the document review and observation of deliberations to confirm, challenge, and refine emerging themes from the interviews. The reliability and validity of the data were enhanced through iterative data collection, the use of multiple methods for data collection, and the ongoing discussion of findings within the research team.

**Table 1:** Data collection methods, sources, and objectives

**Methods (sample size)**	**Sources**	**Objectives**
Group interviews (10) and individual interviews (7)	Face-to-face interviews with knowledge translation platform (KTP) leaders, policymakers and stakeholders and KTPE team members	Solicit in-depth views on activities, outcomes and impacts (if any), and lessons learned, including facilitating factors and challenges
Observation of deliberations (4 plenaries and 6 break-out sessions)	Observation of deliberations at the International Forum on Evidence-Informed Health Policy (EIHP) in Low and Middle Income Countries (LMICs), Addis Ababa, August 28^th^-30^th^ 2012	Describe the climate, activities, and lessons learned, including facilitating factors and challenges
Document review (1 report)	Report from the International Forum on EIHP in LMICs, Addis Ababa, August 28^th^-30^th^ 2012	Identify the climate, outcomes achieved, and lessons learned, including facilitating factors and challenges

A multi-level purposive sampling strategy was used. First, KTPs that were funded by Supporting the Use of Research Evidence (SURE) and the Alliance for Health Policy and Systems Research (AHPSR) were sampled. Second, individuals and groups of individuals were sampled based on their association with these platforms. Third, documents and deliberations held at the Forum were sampled based on their relevance to the work undertaken by the platforms. The specifics of each purposive sampling strategy are detailed in the sections that follow.

Ten KTPs were funded by one or both of SURE and the AHPSR and were included in this study (Table 2). SURE is a collaborative project that supports the EVIPNet in Africa and the Region of East Africa Community Health (REACH) Policy Initiative [11]. The AHPSR’s overall goal is to promote the generation and use of health policy and systems research as a means to improve health and health systems in developing countries. It pursues this goal by developing and harnessing existing methods and approaches to improve both the quality of research and its uptake [12]. Four of these KTPs are WHO-sponsored EVIPNet’s: EVIPNet Burkina Faso, EVIPNet Cameroon, EVIPNet Central African Republic (CAR), and EVIPNet Ethiopia. KTPs funded by the AHPSR include EVIPNet Cameroon and ZAMFOHR, as well as other more project-oriented (e.g., time-limited) initiatives such as Evidence to Policy (E2P) Argentina, E2P Bangladesh, and E2P Nigeria. The Sudan KTP was initially supported by the World Health Organization Regional Office for the Eastern Mediterranean (WHO EMRO). REACH Uganda is a closely aligned initiative with WHO-sponsored KTPs.

**Table 2:** Participating knowledge translation platforms (KTPs) and their host institution

**Participating KTPs**	**KTP affiliates interviewed in the structured reflection**		**Host institution**
	**KTP leaders**	**Policymakers and stakeholders**	
Evidence to Policy (E2P) Argentina	1	-	Non-governmental organization (NGO)
E2P Bangladesh	1	-	Private research institution
E2P Nigeria	3	11	University
EVIPNet Burkina Faso	2	5	Ministry of Health (MOH)
EVIPNet Cameroon	2	1	Hospital
EVIPNet Central African Republic	2	-	University
EVIPNet Ethiopia	4	-	MOH
Regional East African Community Health Uganda	4	-	University
Sudan KTP	1	-	MOH
Zambia Forum for Health Research	3	-	NGO

### Framework for assessing knowledge translation platforms (KTPs)

The framework developed by Lavis et al. [13] was used to guide data collection. The framework identifies four domains for assessing country-level efforts to link research to action: i) the general climate for research use; ii) the production of research that is both highly relevant to and appropriately synthesized for research users; iii) the mix of clusters of activities used to link research to action which include push efforts, efforts to facilitate user-pull, user-pull efforts, and exchange efforts; and iv) the evaluation of efforts to link research to action. The framework also identifies the corresponding elements that are conducive to linking research to action in each domain (Table 3). The elements of this framework are based on potential innovations that are being implemented to link research to action but that require further rigorous evaluation. This framework was previously adapted to assess activities of researchers [14,15] and health research funding agencies in linking research to action in LMICs [16].

**Table 3:** Framework for assessing country-level efforts to link research to action^*^

**Domain**		**Elements**
**General climate**		• Funders, researchers, universities and other research institutions, research users, and intermediary groups support or place value on efforts to link research to action
**Production of research**		• Efforts to engage in priority-setting processes, produce and use scoping reviews, systematic reviews, and single studies when needed
		• Efforts to develop the capacity of researchers to prepare evidence briefs and other forms of research synthesis
**Activities used to link research evidence to action**	**Push efforts**	• Efforts to prepare and communicate evidence briefs to research users
		• Efforts to communicate research findings, which may include identifying actionable messages, fine-tuning messages for different user groups, using evidence-informed strategies to support action based on the messages, and evaluating their impact
		• Efforts to enhance the capacity of researchers to develop and execute evidence-informed push efforts and evaluate their capacity
	**Efforts to facilitate user-pull**	• Efforts to provide access to research (e.g., rapid-response units and ‘one-stop shopping’ to meet users’ needs for high quality research)
		• Efforts by researchers to develop research users’ capacity to use research
	**User-pull efforts**	• Efforts to facilitate research use, such as efforts to assess and enhance the capacity of research users to acquire, assess, adapt, and apply research
		• Efforts to develop structures and processes to help research users to acquire, assess, adapt and apply research; to combine research with other types of information as inputs to decision-making; and to promote the use of research evidence in decision-making
	**Exchange efforts**	• Deliberative processes (such as policy dialogues) and meaningful partnerships between researchers and policymakers to jointly ask and answer relevant questions
		• Efforts to enhance the capacity of researchers and research users to engage in mutually beneficial partnerships
**Evaluation**		• Supporting and participating in rigorous evaluations of efforts to link research to action, outcomes, impacts, and unanticipated consequences
		• Evaluating sustainability (institutionalizing KTPs, governance, structure, function resources, etc.), lessons learned, and opportunities for improvement

^*^ Adapted from Lavis et al. [13].

### Group and individual interviews

Purposive sampling was used to select participants based on their association with these platforms. KTP leaders (and team members), responsible for directing and coordinating activities of the KTP in their own countries, were interviewed. Additionally, policymakers and stakeholders related to the work of these platforms from different settings were interviewed. These were identified via a respondent-driven technique whereby KTP leaders suggested potential key informants who could provide additional information. Interviews were also conducted with members of the KTPE team, which includes researchers from McMaster University, the American University of Beirut, and Pontificia Universidad Catolica de Chile. The KTPE team members are part of the *Evaluating Knowledge-Translation Platforms in Low- and Middle-Income Countries* project that was initiated in 2009 to evaluate KTPs that are being launched around the world.

Participants were notified of the objectives of the interviews and were approached for the interview after securing their verbal approval. All interviews were conducted face-to-face. Interviews were tape-recorded only after obtaining the permission of participants. All interview data was treated as confidential and the anonymity of participants was preserved. Care was taken to ensure that comments could not be attributed to a single individual (or KTP) to protect the confidentiality of respondents.

Ten group interviews were conducted with a total of 39 key informants. Group size ranged from two to eight participants per group. Group interviews with as few as two participants were previously used in several qualitative studies [17-20]. Group interviews lasted for one to two hours on average. In addition, seven individual interviews were conducted and lasted for an average of 45 minutes. Six group interviews were tape-recorded and were transcribed verbatim. Extensive notes were taken for the remaining group interviews and for the individual interviews and they were summarized shortly thereafter.

Interviews with KTP leaders were guided by a semi-structured interview tool developed by the KTPE team. An adapted version of the interview tool was used for interviewing policymakers, stakeholders, and KTPE team members, which allowed them to focus on questions based on their area of expertise. The development of the semi-structured interview tool was guided by the framework adapted from Lavis et al. [13] and involved reviewing several iterations by team members to ensure its robustness. The interviews covered topics on general climate for supporting (or hindering) efforts to link research to action; activities and outputs undertaken by KTPs including priority setting, engaging in push efforts, facilitating user-pull, pull efforts, and exchange efforts; KTP’s evaluation efforts and their perceptions of the results of their activities; facilitators for supporting KTP work, challenges encountered, and lessons learned that can assist in overcoming challenges; and critical factors that can help to ensure the sustainability of KTPs. Interview tools are enclosed in Additional files 1 and 2.

### Document review

The document review helped to identify efforts undertaken by KTPs to link research evidence to action and derive key lessons for improving country-level efforts to support EIHP in LMICs. The document review was cross-checked with findings from interviews and observation of deliberations. Material examined included a report summarizing the proceedings of the International Forum on EIHP in LMICs. The report was prepared by the WHO International Forum Organizing Committee and it provides an overview of the Forum including plenaries, workshops, and small group sessions that took place. It is organized around the framework for assessing country-level efforts to link research to action and highlights key messages and lessons learned that emerged during the Forum [21]. The document was reviewed and summarized in a data collection sheet that included the title of the document and the data related to the four domains for assessing efforts to link research to action framework.

### Observation of deliberations

Observations of deliberations at the International Forum on EIHP in LMICs were recorded. Materials examined included notes on observation of deliberations and hands-on workshops, and meeting minutes of four plenaries and six sessions from the Forum. Sessions from the Forum were sampled based on their relevance to the work undertaken by these KTPs. Participants in these sessions shared their experiences on the ways in which KTPs can encourage and promote EIHP initiatives and engage different actors in efforts to support EIHP. The observations focused on the climate for and activities of KTPs, facilitators for supporting KTP work, challenges encountered, and lessons learned that can assist in overcoming these challenges. Observations were recorded by three members of the research team. They were then compiled and compared against each other. Observations helped to verify information obtained through the interviews and the document review. Each document was reviewed and summarized in a data collection sheet.

### Data analysis

Data analysis was guided by the framework developed by Lavis et al. [13]. Data analysis was conducted by the lead author (FEJ) using a grounded iterative approach, and shared with the other team members (JNL, SC, TP, KAM) during several stages of the analysis as a way to confirm, challenge, and feedback into the further refinement of themes. Preliminary results were also shared with a group of doctoral students studying EIHP to the same effect.

Data from the interviews, document review, and observation of deliberations were holistically analyzed such that data collected from the three sources were gathered using the same data analysis sheet, rather than analyzing data from different sources separately. Thematic analysis was used. Open coding was conducted first, whereby findings were broken into similar concepts and ideas. Axial coding was then conducted, which involves organizing the emerging concepts into themes [22]. Themes were pre-identified based on the study objectives, interview questions, and additional themes emerged during the analysis. Themes were then categorized based on the framework by Lavis et al. [13]. Recurring themes and emerging patterns across respondents from different KTPs and the corresponding countries were then analyzed. Illustrative quotations were identified to support the narrative description of the themes.

## Results

A total of 23 KTP leaders and 17 policymakers and stakeholders were interviewed from the 10 KTPs (Table 2), in addition to 7 KTPE members. At the time of the interviews, KTPs were three years old except for E2P Argentina, E2P Bangladesh, and EVIPNet Ethiopia, which were two years old. KTPs were at different levels of implementation. EVIPNet Burkina Faso and EVIPNet Ethiopia were at the early phases of implementation and were recruiting human resources and planning to expand activities. The remaining KTPs were planning for assessing the outcomes of their activities or for sustaining their KTPs and obtaining long-term funding.

The following sections present the common themes that emerged from the interviews and that were triangulated and confirmed with the document review and observation of deliberations. The themes were grouped under the main domains for assessing country-level efforts to link research to action.

### General climate

The extent of support for and value given to efforts to link research to action was variable among KTPs. Results from the interviews, document review, and observation confirmed that the support provided to KTPs by policymakers and stakeholders as well as the financial support provided by international funders were key for enabling EIHP initiatives (Table 4). The main challenges to supporting EIHP work were the lack of skilled human resources to undertake KT activities and the difficulty in managing multiple roles within KTPs.

**Table 4:** Activities used to link research to action, facilitators, challenges, and lessons learned

**Themes**	** Results**
**Climate**	
*Activities*	• No systematic activities identified although a range of ad hoc activities were undertaken
*Facilitators*	• Policymakers’ and stakeholders’ support
	• International funding support
*Challenges*	• Lack of skilled human resources to undertake knowledge translation (KT) activities
	• Gaps in infrastructure (e.g., lack of a functional internet connection)
*Lessons learned*	• Increase awareness among policymakers and stakeholders including those outside the health sector
	• Increase financial and technical support from funders
**Research production**	
*Activities*	• Three Knowledge Translation Platforms (KTPs) built their capacity for conducting systematic reviews and undertaking priority-setting exercises
	• Six KTPs conducted priority-setting exercises with policymakers to identify high priority policy issues prior to pursuing EIHP activities
	• Two KTPs produced systematic reviews
*Facilitators*	• None identified
*Challenges*	• Lag in or lack of local research production
*Lessons learned*	• Build the production of local evidence
**Push efforts**	
*Activities*	• All KTPs built their capacity to develop evidence briefs for policy
	• All KTPs developed evidence briefs
*Facilitators*	• None identified
*Challenges*	• Lack of skilled human resources to pursue push efforts
	• Poor quality of local information
*Lessons learned*	• Build capacity within KTPs for push efforts
	• Build capacity of researchers to align research with policy and disseminate research on policy-relevant topics
**Facilitate user-pull**	
*Activities*	• Four KTPs are in the process of implementing rapid response services (RRS)
	• Five KTPs are in the process of creating online clearinghouses
*Facilitators*	• None identified
*Challenges*	• Lack of highly skilled and dedicated personnel to provide RRS and one-stop shopping
	• Difficulty in accessing and finding local evidence
*Lessons learned*	• Build capacity of KTPs to undertake such activities
	• Increase financial support to pursue capacity building for such activities
**User-pull**	
*Activities*	• One KTP engaged in efforts to assess and enhance the capacity of research users to acquire, assess, adapt, and apply research
*Facilitators*	• Strong leadership and political will
*Challenges*	• High turnover in top level policymakers in government
	• Resistance to change and strong political influences
*Lessons learned*	• Assess and build capacity among research users to acquire, assess, adapt, and apply research
	• Establish institutional structures and routine processes to support evidence-informed health policymaking (EIHP)
**Exchange**	
*Activities*	• All KTPs engaged in organizing deliberative dialogues informed by evidence briefs
*Facilitators*	• Skilled human resources to moderate deliberative processes
	• Support of policymakers and stakeholders
	• Location within the Ministry of Health brings KTPs closer to policymakers and stakeholders
*Challenges*	• Difficulty in convincing policymakers, stakeholders, and researchers to interact
	• High turnover in top level policymakers in government
*Lessons learned*	• Build the capacity of researchers and KTPs to engage in exchange efforts
	• Extend interactions to community members, donors, international community, and the media
	• Provide incentives for the participation of policymakers and researchers
	• Interact with other KTPs to share experience and best practice
**Evaluation**	
*Activities*	• Four KTPs had evaluated evidence briefs, deliberative dialogues, and capacity building sessions through pre/post intervention questionnaires
	• None of the KTPs had yet begun to assess the long-term impact of their activities in policymaking
*Outcomes*	• Seven KTPs reported increased awareness of the importance of EIHP initiatives among policymakers, stakeholders, and researchers
	• Eight KTPs reported strengthened relationships among policymakers, stakeholders, and researchers
	• Six KTPs reported that their evidence briefs helped inform policymaking at the government level
	• Six KTPs reported increased demand for KT products by policymakers
	• Three KTPs reported enhanced capacity among KTP members for developing evidence briefs and deliberative dialogues
	• One KTP reported enhanced capacity among policymakers for accessing, assessing, and using research evidence
*Facilitators*	• Strong leadership support particularly from policymakers at the government level to achieve outcomes
*Challenges*	• Perception that monitoring and evaluation (M&E) activities were challenging
	• Lack of capacity within KTPs to conduct M&E activities
*Lessons learned*	• Intensify M&E efforts to build the case for sustainability
**Sustainability**	
*Views*	• Six KTPs viewed their work as a long-term initiative
*Facilitators*	• None identified
*Challenges*	• Difficulty in ensuring the sustainability of EIHP initiatives
	• Difficulty in identifying long-term sources of funding
*Lessons learned*	• Institutionalize KTPs within the structures (or processes) of the government
	• Build and retain capacity within KTPs and similar organizations
	• Apply for funding from international and governmental sources or conduct revenue-generating activities such as RRS

Despite efforts by funders, policymakers, and stakeholders to support EIHP, findings suggested that much should be done for building support for EIHP initiatives in LMICs. Increasing the awareness of policymakers and stakeholders on the importance of EIHP, including those outside the health sector, was often highlighted as a requirement for creating a climate that supports KT activities. For example, discussions at the Forum highlighted the importance of mapping institutional structures at the national level to understand the optimal ways and emerging opportunities for targeting policymakers and stakeholders, which would also require that KTPs build their capacity in lobbying and understanding the political context. Additionally, KTP leaders repeatedly urged funders to play a stronger role in supporting KT activities in LMICs mainly through increasing financial support provided to KTPs, strengthening technical capacity of KTPs to undertake KT, as well as providing mentorship opportunities to KTPs and more flexibility with funding (for example in terms of providing KTPs with more time to discuss and explore innovative KT ideas) (Table 4).

### Production of research

Findings revealed that efforts exerted by KTPs under the domain of research production are still limited. Interviews indicated that only a few KTPs engaged in producing systematic reviews. The lag in or lack of local research production and the urgent need to build the production of high quality, policy-relevant systematic reviews and local single studies were repeatedly emphasized across interviews, the document review, and observations (Table 4).

### Push efforts

Push efforts constituted the bulk of KTP efforts to link evidence to policymaking. All KTPs built their capacity on developing evidence briefs for policy and they all developed evidence briefs, with significant variation in the number of produced briefs across KTPs at the time of interviews. A common perception among the interviewed KTPs was that evidence briefs were useful tools for influencing policymaking. This view was illustrated by a KTP leader:

“We have done interesting work in terms of working with policymakers and researchers in developing evidence briefs that could be used for policymaking”

Participants acknowledged that the process of developing evidence briefs was burdensome and lengthy; this was mainly attributed to a lack of skilled human resources to prepare evidence briefs and poor quality of local information to support evidence briefs. As such, the need for further capacity building to ensure that organizations and individuals within countries have the skills to prepare evidence briefs was repeatedly emphasized. Additionally, participants emphasized the need for researchers to better align their research with high priority policy issues in their countries and accelerate their efforts in disseminating research evidence and making it readily accessible for policymaking (Table 4).

### Efforts to facilitate user-pull

Establishing rapid response services (RRS) and “one-stop shopping” through online clearinghouses were undertaken by some KTPs in order to facilitate user-pull, with varying degrees of implementation across KTPs. While challenges encountered under this domain varied across KTPs, a set of common challenges emerged from the interviews and were confirmed by the document review and observations. These common challenges were the lack of skilled and dedicated personnel to conduct efforts to facilitate user-pull and the difficulty encountered by KTPs in accessing and finding local evidence mainly due to the unwillingness of policymakers and research institutions to share local data. As such, the need for building the capacity of KTPs to undertake efforts that facilitate user-pull and for scaling up financial support to pursue capacity building for such initiatives were frequently emphasized (Table 4). REACH Uganda, EVIPNet Burkina Faso, and ZAMFOHR highlighted how collaboration among them helped to develop their skills in implementing RRSs. They emphasized that this type of knowledge sharing is essential for scaling up approaches to facilitating user-pull in other countries.

### User-pull efforts

Efforts to enhance the capacity of research users to acquire, assess, adapt, and apply research were rarely undertaken by KTPs. Common challenges to pursuing user-pull efforts included resistance to change and strong political influences. Additionally, a number of KTPs reported that high turnover in top level policymakers in the government, including political appointees and civil servants, contributed to the leakage of capacity, severed relationships, and shuffled priorities, as a KTP leader explained:

“Top level policymakers will stay for a short period of time because their appointment is political. As such, it is important to target mid-level policymakers because they are the engine of policymaking, stay longest in the system, and can be more readily reached.”

In order to support user-pull efforts, interviews and the document review indicated that there is a need to assess and build capacity among research users to acquire, assess, adapt and apply research to help ensure their active engagement in EIHP initiatives. Such efforts should be combined with establishing institutional structures and routine processes (e.g., criteria for promotion and legislation) for research users to encourage their engagement in EIHP activities. For example, E2P Nigeria contacted policymakers through formal organizations in order to encourage their participation in EIHP initiatives and provided certificates following capacity building sessions (Table 4).

### Exchange efforts

Efforts to engage in deliberative processes and collaboration among research users, researchers, and funders constituted a core component of EIHP initiatives. Deliberative dialogues informed by evidence briefs were perceived by interviewees as the “most commendable [tools for enhancing EIHP]” and “activities [KTPs] were most proud of”. Interviews reported that deliberative dialogues had the potential to influence the use of evidence in policy formulation, influence the perceptions of policymakers, stakeholders, and researchers regarding the availability of research evidence, and strengthen the relationship between policymakers, stakeholders, and researchers. At the same time, interviewees emphasized that these are their own perceptions about the short-term influence of deliberative dialogues and that it is still early in their KTP work to comment on the long-term impact of deliberative dialogues on the use of evidence in policymaking. These perceptions are illustrated in the following quotes by KTP leaders:

*“Deliberative dialogue was the main pathway of influence. It provided a public face to our work.*”

*“Options that were proposed* [in the deliberative dialogue informed by an evidence brief] *were integrated in the strategic plan for accelerating maternal survival.”*

*“*[During deliberative dialogues] *it was very interesting to see how actors with different interests and views on the healthcare system were willing to discuss new ideas, and contribute without putting aside their differences. This was very encouraging for us.”*

Findings revealed that key components that facilitated the engagement of KTPs in conducting deliberative processes were skilled human resources to moderate deliberative processes and the support of policymakers and stakeholders and their willingness to participate in such initiatives. In addition, being hosted within the Ministry of Health (MOH) facilitated interactions between KTPs and policymakers and stakeholders.

Suggestions for strengthening exchange efforts included extending interactions beyond those between researchers and policymakers at the government level to include a diverse range of research users including community members, donors and the international community, and the media. Increasing opportunities to engage with other KTPs doing similar work in order to share experience and best practice was also highlighted, as a KTP leader stated:

“It would have been interesting to have more interaction with other KTPs in order to see how other initiatives are working and whether they face similar issues and how they address them.”

### Evaluation

A common theme that emerged from the interviews and was confirmed by the document review and observations was the need for intensifying monitoring and evaluation (M&E) efforts. Generally, M&E activities were seldom undertaken by KTPs in LMICs. This lag in conducting M&E activities was attributed to the lack of capacity within KTPs to conduct M&E activities, particularly in implementing M&E approaches and tools, and in analyzing data, as well as the prevailing perception among KTP leaders that M&E activities were particularly challenging endeavors.

When asked about the key outcomes resulting from EIHP activities, interviewees emphasized the increased awareness among policymakers, stakeholders, and researchers on the importance of EIHP initiatives. This was illustrated by a policymaker:

*“*[The KTP in our country] *has become a household name in the MOH as evident by the level of participation* [of policymakers and stakeholders] *in EIHP initiatives.”*

Additionally, strengthened relationships among policymakers, stakeholders, and researchers were common outcomes of the KTP work, as mentioned in the interviews. Furthermore, evidence briefs reportedly helped inform policymaking at the government level. For example, policymakers from Nigeria attested to the utility of evidence briefs developed by E2P Nigeria in informing decisions for launching the Ebonyi State Helminth Control Programme. Other frequently mentioned outcomes included an increased demand for KT products by policymakers, enhanced capacity among policymakers for accessing, assessing, and using research evidence, and enhanced capacity among KTP members for developing evidence briefs and dialogues.

Key to achieving outcomes and bringing about change was strong leadership support particularly from policymakers at the government level, as emphasized in the interviews, document review, and observation of deliberations. Discussions during the interviews and at the Forum of key facilitators for conducting KTP work acknowledged the central role that strong leadership plays in the process of establishing collaborative partnerships between research funders, research producers and users, and for promoting EIHP initiatives. KTP leaders reported that committed high-level policymakers within the government (e.g., advisors of the Minister of Health) were among the essential facilitators to their KT work.

### Sustainability

Ensuring the sustainability of EIHP initiatives after the end of funding was identified by interviewees as a major challenge confronting KTPs. Three interrelated factors were commonly cited to play a strong role in influencing the sustainability of KTPs.

The first factor that emerged was the institutionalization of KTPs within the structures (or processes) of the government. There were various arrangements for hosting KTPs. Three KTPs were hosted by the MOH, two were hosted by a university, one by a government research institution, and one by a hospital, while one was an independent non-governmental organization (NGO) (Table 2). Findings on the structure, pros, and cons of the different KTP arrangements are summarized in Table 5.

**Table 5:** Key features of different arrangements of Knowledge Translation Platforms (KTPs)

	**Structure**	**Pros**	**Cons**	**Examples**
Ministry of Health (MOH)	• KTP as a permanent structure within the MOH	• Activities and products address priority needs of government	• Challenges in managing political pressures in the work of the KTP	• EVIPNet Burkina Faso
				• EVIPNet Ethiopia
			• Policymakers in high MOH positions are seldom available for meetings	
				• Sudan KTP
		• Closer and more permanent relationships with the government		
			• Restricted network of researchers	
			• Difficulty engaging lower level policymakers and local leaders	
		• Integration with MOH structure and function strengthens the sustainability of the KTP		
	• Researchers are commissioned by the KTP			
University/private research institution	• KTP located within university/private research institute	• Minimal political influence and more independence of KTP work	• KTP members not permanently available for KTP work, as they have different jobs	• E2P Nigeria
				• EVIPNet Central African Republic
				• Regional East African Community Health (REACH Uganda)
				• E2P Bangladesh
			• Difficulty sustaining KTP due to funding and lack of institutionalization	
	• KTP composed mainly of researchers, policymakers and other members from the MOH and civil society			
Non-governmental organization (NGO)	• KTP established as an independent NGO	• Minimal political influence and more independence of KTP work	• Difficulty sustaining KTP due to funding and lack of institutionalization	• E2P Argentina
				• Zambia Forum for Health Research
	• Researchers commissioned by the KTP			
Hospital	• KTP located in the hospital	• Minimal political influence and more independence of KTP work	• KTP members not permanently available for KTP work, as they are recruited by hospital for conducting other tasks	• EVIPNet Cameroon
	• KTP members paid by hospital and conduct KTP work on part-time basis			
			• Sustainability challenges of KTP due to limited funding	

Institutionalization was thought to help overcome challenges related to retaining capacity and funding, as indicated by interviewees, document review, and observation of deliberations. For example, institutionalization of KTPs within the MOH ensured the continuous funding of EIHP activities and salaries. This can be demonstrated by EVIPNet Ethiopia, which was established by the National MOH as the Directorate of Technology Transfer & Research within the EHNRI; it is composed of staff paid by autonomous public authorities to conduct KTP work. Its strategic direction, activities, and KT priorities are informed by policymakers at the MOH. Participants noted that close linkages to the MOH strengthens the sustainability of KTP work, provides proximity to policymakers, and increases the prospects that KTP work is utilized in policymaking.

At the same time, it was emphasized that KTPs should pay attention to maintaining their autonomy from political interests and governmental control and should protect themselves from the instability and turnover in top level policymakers at the government level, which are mainly influenced by political changes in authority. Being located within a university or a research institute whilst having close collaboration with and support from policymakers and stakeholders was a suggested alternative for institutionalization:

*“The instability in the MOH and lack of appropriate capacity makes the university a better alternative* [for our KTP]*.”*

The second factor influencing sustainability was funding availability*.* All KTPs were financed by international funders (e.g., SURE and AHPSR) and six of these reported difficulty in identifying long-term sources of financing following the end of international funding, as a KTP leader illustrated:

“Lack of long-term funding means that efforts are focused on looking for funding instead of focusing on implementing activities effectively.”

Finally, the third key factor that emerged to influence sustainability was the need for building and retaining capacity within KTPs, with special focus on conducting M&E activities and developing skills in advocacy and “selling the idea” of EIHP (Table 4).

## Discussion

### Principal findings

The establishment of KTPs in a broad range of LMICs is a promising development in supporting EIHP. KTPs report that they have contributed to increased awareness of the importance of EIHP initiatives and strengthened relationships among policymakers, stakeholders, and researchers, while acknowledging the urgent need for KTPs to conduct more robust M&E efforts to assess the outcome and impact of their activities on health policymaking. Furthermore, deliberative dialogues informed by evidence briefs for policy have been among the most commended tools by interviewees for informing policymaking, partly because they provided a public face for the work of KTPs. Additionally, deliberative dialogues can potentially strengthen interactions among policymakers, stakeholders, and researchers, which in turn increases the prospects for research use in policymaking [23].

It is important to note that KTPs were not able to specify how research evidence was used in policymaking (e.g., was the use of evidence instrumental, conceptual, or symbolic? Did it inform policy development or implementation?). The impact of implementing evidence-informed policymaking (e.g., improvement on population health status) has not been assessed and there were no clear mechanisms for undertaking such initiatives. However, this structured reflection provides a snapshot in time of the work of KTPs and their efforts to scale up activities to link research evidence to policymaking and assess the impact of their work, which is still ongoing.

Findings from this structured reflection indicated that KTPs have scaled up push and exchange efforts to link research evidence to policymaking. However, KTPs’ production of research, efforts to facilitate user-pull, user-pull efforts, and evaluation efforts need to be intensified in their view. In order to facilitate these efforts, findings suggested building the production of local evidence, building the capacity of KTPs and research users to undertake such activities, and increasing financial support to pursue capacity building and M&E activities.

Variations in achieving EIHP activities among KTPs have been due to the differences in support systems and value placed on efforts to link research to action available to KTPs in their countries. Strong leadership and support from policymakers at the government level in addition to funding and technical support (e.g., availability of mentors) from international funders were key facilitators to conducting EIHP activities. However, the lack of skilled and dedicated human resources to conduct push efforts, efforts to facilitate user-pull and exchange efforts, and to evaluate these efforts impeded KTPs.

Ensuring the sustainability of EIHP initiatives after the end of funding was identified as a major challenge confronting KTPs. KTPs reported that institutionalization within government structures or processes has helped to retain human resources and secure funding for their KTP activities, whereas KTPs hosted by universities highlighted the advantage of attaining autonomy from political interests while maintaining close collaborations with and support from policymakers and stakeholders.

### Strengths and limitations

The study has several strengths. First, the data were collected using three different sources (interviews, document review, observation of deliberations), which helped enhance understanding of KTP experiences and allowed cross-checking of findings. Second, the “framework for assessing country-level efforts to link research to action” that was used for analysis helped build a comprehensive understanding of activities, the set of facilitators and challenges influencing each of these activities, and key learnings for enhancing these activities within each component of the framework. Third, this study is one of the very few reporting on the early experiences of KTPs in LMICs and can help identify opportunities for improving ongoing activities of KTPs and informing the experiences of other growing KTPs in LMICs.

With regards to limitations, our sample of interviewees was drawn from KTP staff and leaders and then from policymakers and stakeholders. Eliciting the opinion of a larger number of policymakers and stakeholders at the country level can potentially provide additional in-depth insights on the work of KTPs from the perspective of the groups that KTPs eventually aim to reach and influence. This can provide valuable information for assessing initiatives conducted by KTPs, tailoring EIHP initiatives to their needs, and improving the work of KTPs in each setting. That said, KTP leaders come from different backgrounds, such as policymakers at the government level and stakeholders from civil society as well as researchers, and have provided their perceptions on EIHP activities from these perspectives. Additionally, the document review and observations and synthesis of deliberations from the International Forum included findings from the perspectives of policymakers, stakeholders, and researchers, in addition to KTP leaders.

### Findings in relation to previous studies

Findings from this study on the barriers and facilitators to KTP work are congruent with those previously reported by policymakers, stakeholders, and researchers on EIHP, particularly from LMICs [7,24-30]. Gaps in research production and limited alignment with regional priorities, as well as lack of skills among research users to acquire, assess, adapt, and use research evidence, were also previously reported to hinder EIHP activities in LMICs [25-28]. There is little empirical evidence on effective KT approaches and how KT strategies can be tailored for different contexts and disciplines [31-35]. The positive views held by interviewees regarding evidence briefs and deliberative dialogues are supported by a recent survey of policymakers, stakeholders, and researchers from LMICs, whereby respondents viewed evidence briefs and deliberative dialogues very favorably and reported strong intentions to act on what they learned in the briefs and dialogues [36]. Lessons drawn from the experience of ZAMFOHR included the necessity of performing comprehensive situation analyses to understand the evidence-to-policy climate and the operational niche for the KTP and the importance of networking to benefit from the experience of other KTPs, funding opportunities, and technical support [7]. ZAMFOHR’s experience, as well as findings from other studies, suggested focusing the KTP program on building the capacity of its own staff, policymakers, stakeholders, and researchers to engage in EIHP [7,26-29], which also confirm lessons learned from KTPs in the current study. Furthermore, making changes to the existing institutional structures and incentives provided to researchers and research users were suggested strategies to encourage them to engage in EIHP initiatives [25,28-30].

### Implications for practice and future research

Findings from this study suggest the need for intensifying efforts to increase research on effective KT strategies and understanding KT tools in different contexts. Specifically, further research is needed to systematically determine how the ways in which evidence briefs, deliberative dialogues, and other KT strategies are designed, their content and the context in which they are developed will influence their usefulness in supporting EIHP [9,36,37]. A recent systematic review reported that contextual factors, particularly the institutions, interests, and values of a given context, as well as issue-related factors (such as whether issues are polarizing, salient, or familiar to policy actors) can influence views regarding evidence briefs among their intended users [38]. Similarly, future research may explore the ways in which contextual factors and policy issues can influence other strategies (e.g., capacity building targeted at research producers and users) for supporting the use of research evidence in policymaking processes.

There is a general assumption that evidence-informed policymaking will inevitably improve outcomes; however, there is no robust evidence base to support/disprove this assumption. As such, assessing the impact of policies informed by evidence briefs for policy and deliberative policy dialogues (e.g., improved health of the population) is needed to complete the M&E cycle and to make the case for supporting EIHP. Further research comparing governance structures of KTPs in different contexts and the factors influencing their sustainability is also much required, given the current volatility of these initiatives as indicated in this study.

This structured reflection provided four key lessons to improve the activity of KTPs and to help inform those interested in establishing KTPs in their own countries. Firstly, concerted efforts should be directed towards capacity building for preparing evidence briefs, conducting deliberative dialogues, implementing a RRS, developing and maintaining online clearinghouses, and evaluating these activities among those engaging in these EIHP activities. A systematic review reported that training in using systematic reviews and providing peer-group support facilitated the uptake of evidence from systematic reviews [31]. Capacity building ought to go hand-in-hand with raising the awareness of policymakers, stakeholders, and researchers alike on the importance of EIHP in addition to developing their skills to engage in EIHP activities (e.g., skills of policymakers to access, assess, and apply research; and skills of researchers to align research production with policy priorities, produce systematic reviews, effectively disseminate their research, and engage with policymakers). In Nigeria, for example, a training workshop that focused on capacity development for EIHP and building partnerships between policymakers, stakeholders, and researchers showed significant improvements in participants’ knowledge and their understanding of the health policymaking process and the use of evidence [8]. Different capacity-building approaches can be implemented and assessed with both researchers and research users and tailored to different contexts; for example, comparing the use of group workshops with one-to-one meetings on building the capacity of policymakers, stakeholders, and researchers [39].

Additionally, funders are in a position to move the agenda of EIHP forward by providing training on KT skills and mentoring or coaching for KTPs, policymakers, stakeholders, and researchers as well as by advocating for KT through rewarding/celebrating the work of those promoting KT [40]. At the same time, such efforts should be combined with establishing institutional structures and incentives that support EIHP activities (e.g., criteria for promotion or legislation to mandate the use of research evidence). A systematic review on the use of research evidence in public health policymaking suggested that changing the culture within which policymakers work (in terms of structures, rewards, and training) such that more value is placed on the use of research evidence might encourage its use [41]. For example, the Ministry of Health and Long-Term Care in Ontario requires training for civil servants in finding and using research evidence, incorporating assessments of the use of research evidence as part of performance reviews. It also requires civil servants making submissions to the minister or to cabinet to document the key sources for research evidence that were searched and declare that relevant findings were used to inform the submission [42].

Secondly, the further expansion of financial support from funders, government sources, or revenue-generating activities will be needed to support and retain capacity within KTPs as well as to ensure the sustainability of these activities. Additionally, funding agencies could play a stronger role in promoting EIHP by requiring the presence of a KT component as a condition for the funding of proposals and by facilitating the sharing of experiences across different KTPs and international and local actors. For example, the Michael Smith Foundation for Health Research proposes five key functional areas that funders can undertake for effective KT, these are advancing KT science, building KT capacity, managing KT projects, funding KT activities, and advocating for KT [40]. This funding agency suggests specific examples of funding agencies’ activities, including funding knowledge synthesis and other KT strategies as well as KT model testing, providing awards for research use and uptake (e.g., to adapt and implement research evidence), and developing partnerships with governmental organizations and NGOs in order to leverage limited human and financial resources and align with existing and new initiatives and research [40].

Thirdly, there is a need for each KTP to think strategically about institutionalizing itself in a way that provides greater access to support and a better opportunity to influence policies. Where the KTP locates itself is a critical variable in its organization and operations and should be based on a comprehensive situational analysis of contextual factors related to EIHPs [7]. It should also be aligned with the structures (or processes) of the government. Additionally, more attention needs to be given to ensuring leaders at all levels with a vision to support EIHP are identified and supported in their work.

Finally, more attention needs to be paid to integrating M&E activities as key components of KTPs right from their conception. M&E activities should be viewed as tools for obtaining the buy-in of research users and funders for supporting KTPs where appropriate, which in turn would help to ensure the sustainability of KTPs. It is important that KTPs integrate their M&E work starting from the planning phase of KT work and that they allocate sufficient resources to implementing M&E. The KTPE team developed a range of tools that can assist KTPs in conducting M&E activities including survey tools to evaluate the perceptions of users of evidence briefs and deliberative dialogues, a brief survey administered every two years to a sample of policymakers, stakeholders, and researchers to assess outcomes of KT activities, and case studies to capture impacts of KT activities on the use of research evidence in policymaking. Very few systematic reviews examine implementing research findings into policy [41]. There are various challenges to evaluating the impact of research use in policymaking; these include metrics that are difficult to define, the fact that the use of research evidence differs depending upon the context, and the resource- and time-intensive nature of impact studies [40]. As such, further research can concentrate on assessing the outcomes and impacts of conducting KT activities on the use of research in policymaking in addition to assessing the impact of evidence-informed policies on the health system and the health of the population.

## Conclusions

Real-time lesson-drawing from the experiences of KTPs can support improvements in the functioning of KTPs in the short term, while making the case for sustaining their work in the long term. Furthermore, the lessons derived from this study can be used to promote the establishment of similar evidence-to-policy initiatives in other countries.

## Abbreviations

AHPSR: Alliance for Health Policy and Systems Research; CAR: Central African Republic; E2P: Evidence to policy; EIHP: Evidence-informed health policymaking; EHNRI: Ethiopian Health and Nutrition Research Institute; KT: Knowledge translation; KTP: Knowledge translation platform; KTPE: Knowledge translation platform evaluation; LMICs: Low- and middle-income countries; M&E: Monitoring and evaluation; NGO: Non-governmental organization; REACH: Region of East Africa Community Health; RRS: Rapid response services; SURE: Supporting the use of research evidence; WHO EMRO: World Health Organization Regional Office for the Eastern Mediterranean; WHO EVIPNet: World Health Organization Evidence-Informed Policy Networks; ZAMFOHR: Zambia Forum for Health Research.

## Competing interests

The authors declare that they have no competing interests.

## Authors’ contributions

All authors meet criteria for authorship. All authors approved the article for submission. FEJ contributed to the conception, design, interpretation of the data, and analysis as well as to drafting and critically revising the article. JNL contributed to the conception, design, and analysis as well as critically revising the article. KAM and TP contributed to the conception, data collection and to critically revising the article. NA contributed to data analysis and reviewing drafts.

## Supplementary Material

Additional file 1 Interview guide (KTP leader version). Click here for file

Additional file 2 Interview guide (policymaker/stakeholder/researcher version). Click here for file

## References

[B1] World Health Assembly Resolution on Health Research 2005 http://www.who.int/rpc/meetings/58th_WHA_resolution.pdf

[B2] World Health Organization The Bamako Call to Action on Research for Health 2008 http://www.who.int/gb/ebwha/pdf_files/EB124/B124_12Add2-en.pdf

[B3] Beijing Statement from the Second Global Symposium on Health Systems Research 2012 http://healthsystemsresearch.org/hsr2012/images/stories/downloads/beijing%20_statement.pdf

[B4] Hamid M Bustamante-Manaog T Truong VD Akkhavong K Fu H Ma Y Zhong X Salmela R Panisset U Pang T EVIPNet: translating the spirit of Mexico Lancet 2005 366 1758 60 10.1016/S0140-6736(05)67709-4 16298204

[B5] Lavis JN Permanand G Oxman AD Lewin S Fretheim A SUPPORT Tools for evidence-informed health Policymaking (STP) 13: preparing and using policy briefs to support evidence-informed policymaking Health Res Policy Syst 2009 7 Suppl 1 13 10.1186/1478-4505-7-S1-S13 20018103 PMC3271824

[B6] Lavis JN Boyko JA Oxman AD Lewin S Fretheim A SUPPORT Tools for evidence-informed health Policymaking (STP) 14: organising and using policy dialogues to support evidence-informed policymaking Health Res Policy Syst 2009 7 Suppl 1 14 10.1186/1478-4505-7-S1-S14 20018104 PMC3271825

[B7] Kasonde J Campbell S Creating a knowledge translation platform: nine lessons from the Zambia Forum for Health Research Health Res Policy Syst 2012 10 31 10.1186/1478-4505-10-31 23034056 PMC3491024

[B8] Uneke CJ Ezeoha AE Ndukwe CD Oyibo PG Onwe F Promotion of evidence-informed health policymaking in Nigeria: bridging the gap between researchers and policymakers Glob Public Health 2012 7 750 65 10.1080/17441692.2012.666255 22394290

[B9] Orem JN Mafigiri DK Marchal B Ssengooba F Macq J Criel B Research, evidence and policymaking: the perspectives of policy actors on improving uptake of evidence in health policy development and implementation in Uganda BMC Public Health 2012 12 109 10.1186/1471-2458-12-109 22316003 PMC3305540

[B10] Young J Research, policy and practice: why developing countries are different J Int Dev 2005 17 727 734 10.1002/jid.1235

[B11] World Health Organization (WHO) Supporting the Use of Research Evidence (SURE) http://www.who.int/evidence/sure/en/

[B12] Alliance for Health Policy and Systems Research (AHPSR) http://www.who.int/alliance-hpsr/about/en/

[B13] Lavis JN Lomas J Hamid M Sewankambo NK Assessing country-level efforts to link research to action Bull World Health Organ 2006 84 620 628 10.2471/BLT.06.030312 16917649 PMC2627430

[B14] Lavis JN Guindon GE Cameron D Boupha B Dejman M Osei EJ Sadana R Research to Policy and Practice Study Team Bridging the gaps between research, policy and practice in low- and middle-income countries: a survey of researchers CMAJ 2010 182 E350 E361 10.1503/cmaj.081164 20439449 PMC2882466

[B15] Cameron D Lavis JN Guindon EG Akhtar T Posada FB Ndossi GD Boupha B Research to Policy and Practice Study Team Bridging the gaps among research, policy and practice in ten low- and middle-income countries: development and testing of a questionnaire for researchers Health Res Policy Syst 2010 8 4 10.1186/1478-4505-8-4 20205837 PMC2825187

[B16] Cordero C Delino R Jeyaseelan L Lansang MA Lozano JM Kumar S Moreno S Pietersen M Quirino J Thamlikitkul V Welch VA Tetroe J ter Kuile A Graham ID Grimshaw J Neufeld V Wells G Tugwell P Funding agencies in low- and middle-income countries: support for knowledge translation Bull World Health Organ 2008 86 524 534 10.2471/BLT.07.040386 18670664 PMC2647493

[B17] Herbert DL Loxton D Bateson D Weisberg E Lucke JC Challenges for researchers investigating contraceptive use and pregnancy intentions of young women living in urban and rural areas of Australia: face-to-face discussions to increase participation in a web-based survey J Med Internet Res 2013 15 e10 10.2196/jmir.2266 23337208 PMC3636288

[B18] Irvine-Meek J Gould ON Wheaton H Todd LE Acceptability and face validity of a geriatric self-medication assessment tool Can J Hosp Pharm 2010 63 225 232 22478982 10.4212/cjhp.v63i3.918 PMC2901782

[B19] Perry JA Olshansky EF A family’s coming to terms with Alzheimer’s disease West J Nurs Res 1996 18 12 28 10.1177/019394599601800102 8686288

[B20] Gregory SW Jr Webster S A nonverbal signal in voices of interview partners effectively predicts communication accommodation and social status perceptions J Pers Soc Psychol 1996 70 1231 40 8667163 10.1037//0022-3514.70.6.1231

[B21] EVIPNet Report on International Evidence Informed Health Policy in Low- and Middle-Income Countries 2012 Addis Ababa, Ethiopia http://global.evipnet.org/wp-content/uploads/2013/02/Addisreport2012.pdf

[B22] Kendall J Axial coding and the grounded theory controversy West J Nurs Res 1999 21 743 757 11512211 10.1177/019394599902100603

[B23] Lavis J Davies H Oxman A Denis JL Golden-Biddle K Ferlie E Towards systematic reviews that inform health care management and policy-making J Health Serv Res Policy 2005 10 Suppl 1 35 48 10.1258/1355819054308549 16053582

[B24] El-Jardali F Makhoul J Jamal D Ranson MK Kronfol NM Tchaghchagian V Eliciting policymakers’ and stakeholders’ opinions to help shape health system research priorities in the Middle East and North Africa region Health Policy Plan 2010 25 15 27 10.1093/heapol/czp059 19948770

[B25] El-Jardali F Jamal D Ataya N Jaafar M Raouf S Matta C Michael S Smith C Health policy and systems research in twelve Eastern Mediterranean countries: a stocktaking of production and gaps (2000–2008) Health Res Policy Syst 2011 9 39 10.1186/1478-4505-9-39 21978482 PMC3224558

[B26] El-Jardali F Lavis JN Ataya N Jamal D Use of health systems and policy research evidence in the health policymaking in eastern Mediterranean countries: views and practices of researchers Implement Sci 2012 7 2 10.1186/1748-5908-7-2 22236561 PMC3286421

[B27] El-Jardali F Ataya N Jamal D Jaafar M A multi-faceted approach to promote knowledge translation platforms in Eastern Mediterranean countries: climate for evidence-informed policy Health Res Policy Syst 2012 10 15 10.1186/1478-4505-10-15 22559007 PMC3445832

[B28] El-Jardali F Lavis JN Ataya N Jamal D Ammar W Raouf S Use of health systems evidence by policymakers in eastern Mediterranean countries: views, practices, and contextual influences BMC Health Serv Res 2012 12 200 10.1186/1472-6963-12-200 22799440 PMC3476435

[B29] Hamel N Schrecker T Unpacking capacity to utilize research: a tale of the Burkina Faso public health association Soc Sci Med 2011 72 31 38 10.1016/j.socscimed.2010.09.051 21074923

[B30] Dowdy DW Pai M Bridging the gap between knowledge and health: the epidemiologist as Accountable Health Advocate (“AHA!”) Epidemiology 2012 23 914 918 10.1097/EDE.0b013e3182605843 23038116

[B31] Wallace J Byrne C Clarke M Making evidence more wanted: a systematic review of facilitators to enhance the uptake of evidence from systematic reviews and meta-analyses Int J Evid Based Healthc 2012 10 338 346 10.1111/j.1744-1609.2012.00288.x 23173658

[B32] Wallace J Nwosu B Clarke M Barriers to the uptake of evidence from systematic reviews and meta-analyses: a systematic review of decision makers’ perceptions BMJ Open 2012 2 e001220 22942232 10.1136/bmjopen-2012-001220 PMC3437427

[B33] LaRocca R Yost J Dobbins M Ciliska D Butt M The effectiveness of knowledge translation strategies used in public health: a systematic review BMC Public Health 2012 12 751 10.1186/1471-2458-12-751 22958371 PMC3532315

[B34] Scott SD Albrecht L O’Leary K Ball GD Hartling L Hofmeyer A Jones CA Klassen TP Kovacs Burns K Newton AS Thompson D Dryden DM Systematic review of knowledge translation strategies in the allied health professions Implement Sci 2012 7 70 10.1186/1748-5908-7-70 22831550 PMC3780719

[B35] Barwick MA Schachter HM Bennett LM McGowan J Ly M Wilson A Bennett K Buchanan DH Fergusson D Manion I Knowledge translation efforts in child and youth mental health: a systematic review J Evid Based Soc Work 2012 9 369 95 10.1080/15433714.2012.663667 22830938 PMC3534353

[B36] Moat KA Lavis JN Clancy SJ El-Jardali F Pantoja T Knowledge Translation Platform Evaluation Study Team Evidence briefs and deliberative dialogues: perceptions and intentions to act on what was learnt Bull World Health Organ 2014 92 20 28 10.2471/BLT.12.116806 24391297 PMC3865546

[B37] Dobbins M Hanna SE Ciliska D Manske S Cameron R Mercer SL O’Mara L DeCorby K Robeson P A randomized controlled trial evaluating the impact of knowledge translation and exchange strategies Implement Sci 2009 4 61 10.1186/1748-5908-4-61 19775439 PMC2936828

[B38] Moat KA Lavis JN Abelson J How contexts and issues influence the use of policy-relevant research syntheses: a critical interpretive synthesis Milbank Q 2013 91 604 48 10.1111/1468-0009.12026 24028700 PMC3790526

[B39] Waqa G Mavoa H Snowdon W Moodie M Nadakuitavuki R Mc Cabe M Swinburn B Participants’ perceptions of a knowledge-brokering strategy to facilitate evidence-informed policy-making in Fiji BMC Public Health 2013 13 725 10.1186/1471-2458-13-725 23919672 PMC3765184

[B40] Holmes B Scarrow G Schellenberg M Translating evidence into practice: the role of health research funders Implement Sci 2012 7 39 10.1186/1748-5908-7-39 22531033 PMC3420241

[B41] Orton L Lloyd-Williams F Taylor-Robinson D O’Flaherty M Capewell S The use of research evidence in public health decision making processes: systematic review PLoS One 2011 6 e21704 10.1371/journal.pone.0021704 21818262 PMC3144216

[B42] Wilson M Lavis J Grimshaw J Supporting the use of research evidence in the Canadian health sector Healthcare Q 2012 15 58 62 10.12927/hcq.2013.23148 24863120

