# Supplementary material for: Capturing lessons learned from evidence-to-policy initiatives through structured reflection
Source: Health Res Policy Syst. 2014 Jan 17;12:2. doi: 10.1186/1478-4505-12-2 (PMC3904410; doi:10.1186/1478-4505-12-2)
Supplement: Additional file 2 — Interview guide (policymaker/stakeholder/researcher version). [file 1478-4505-12-2-S2.doc]

**Additional file 2. Interview guide (policymaker / stakeholder / researcher version)**

1. How familiar are you with the work of the KT platform (use local name)?
2. What **activities/outputs** are you aware of and which were the most useful and why?

- Policy briefs?
- Policy dialogues?
- Priority setting?
- Other?

1. Can you give one or two examples of the KT platform’s **achievements**?

- Outcomes?
  - Do they relate to the availability of relevant research evidence, the strength of relationships between policymakers and researchers, policymakers’ capacity to find and use research evidence or other outcomes?
  - What were the pathways through which the KT platform had an influence on these outcomes?
  - What factors helped or hindered its achievement of the outcomes?
- Impact?
  - Do they relate to the use of research evidence in policymaking and if so how did the work intersect with policymaking and policy implementation processes?
  - What were the pathways through which the KT platform had an influence on this impact measure?
  - What factors helped or hindered your achievement of the outcomes?
- Any unanticipated consequences?

1. What have been the main **barriers** that the KT platform has had to deal with (in general and, where applicable, for specific activities/outputs)?

- Time (not enough time to have an impact)?
- Money (not enough resources, rigid budget categories, delays in releasing funds)?
- Project plan (not enough flexibility)?
- Location (university, government or in between)?
- Team (lack of a common understanding across team, lack of KT training for those who do the work, timing of training in relation to the work)?
- Turn-over (staff, target audience, Alliance)?
- Health system features?
- Political system features?
- Other?

1. What have been the main **facilitators** for the KT platform (in general and, where applicable, for specific activities/outputs)?

- Money (Alliance, other sources)?
- Technical support (training workshops, on-site visits, telephone consultations)?
- SURE meeting (not including the training component per se)?
- Network of people doing similar work (SURE teams, EVIPNet)?
- M&E fellows
- Health system features?
- Political system features?

1. How do you and your organization **feel** about this work?

- Success?
- Learning opportunity?
- Sustainable?
- What are you most proud of?
- What have been the key learnings?

1. How could this work have been a **better experience**?

- More flexibility with the project?
- More groups / activities in a single country?
- More synergies with the Alliance program of work and with other developments (e.g., SURE, CHEPSAA)

1. Will the work **continue** after funding ends and how?

- Funding (government, activities that would raise revenue)?
- Organizational home?
- Continuing professional development?
- Other supports?

1. Any **other reflections**?
